# Three-decade assessment of dry and wet spells change across Iran, a fingerprint of climate change

**DOI:** 10.1038/s41598-023-30040-0

**Published:** 2023-02-18

**Authors:** Armita Motamedi, Alireza Gohari, Ali Torabi Haghighi

**Affiliations:** 1 Department of Irrigation, College of Agriculture, Isfahan University of Technology, Isfahan, 84156-83111 Iran; 2 Water, Energy and Environmental Engineering Research Unit, University of Oulu, P.O. Box 4300, 90014 Oulu, Finland

**Keywords:** Climate sciences, Climate change, Hydrology

## Abstract

Extended periods of hydro-climate extremes with excessive or scarce rainfall associated with high or low temperatures have resulted in an imbalanced water cycle and inefficient socio-economic systems in several regions of Iran. However, there is a lack of comprehensive investigations on short-term to long-term variations in timing, duration, and temperature of wet/dry spells. This study bridges the current gap through a comprehensive statistical analysis of historical climatic data (1959–2018). Results indicated that the negative tendency of the accumulated rainfall (− 0.16/ − 0.35 mm/year during the past 60/30 years) in 2- to 6-day wet spells had made significant contributions to the ongoing downward trend in annual rainfall (− 0.5/ − 1.5 mm/year during the past 60/30 years) owing to a warmer climate condition. Warmer wet spells are likely responsible for precipitation patterns changes in snow-dominated stations since their wet spells temperature has more than threefold growth with increasing distance to coasts. The most detected trends in climatic patterns have started in the last two decades and become more severe from 2009 to 2018. Our results confirm the alteration of precipitation features across Iran due to anthropogenic climatic change, and suggest expected increase in air temperature would likely result in further dry and warm conditions over the coming decades.

## Introduction

Given its substantial role in the hydrological cycle, minor changes in precipitation can significantly affect water, energy, and food security^1^. Moreover, spatiotemporal alterations in precipitation patterns can lead to hydro-climate extremes such as floods or droughts, which are associated with devastating impacts on socio-ecological systems^2^. To address this concern, a great deal of attention has been paid to the investigation of any changes or trends in the main characteristics of extreme precipitation, including extreme rainfall intensity^3–6^, heavy rainfall frequency^7–9^, extreme rainfall magnitude^10,11^, and wet/dry spells^12–14^.

A dry spell is defined as a sequence of dry days that have contributed significantly to the intensity and duration of droughts^15–17^. A wet spell is defined as consecutive days of precipitation. The analysis of dry and wet spell characteristics (i.e., occurrence and duration) provides insight into sustainable hydro-environmental planning and management. Several attempts have been made to quantify spatiotemporal changes in wet and dry spells as well as the associated alterations in precipitation pattern^18–25^. For instance, the spatiotemporal analysis of precipitation patterns in India has indicated that the duration of the maximum dry/wet spell had changed significantly (having either increased or decreased), while no signs of change were found in total precipitation events from 1906 to 2010^26^. The spatiotemporal analysis of dry spells in Iran revealed that dry spells were relatively short and frequent in the northern half of Iran and longer and less frequent in the southern half, while most of the stations under study were at risk of experiencing droughts^27^.

While much of the research up to now has been mainly focused on the duration and number of dry/wet spells^13,18,28–31^, changes in their timing have been poorly explored. Arid and semi-arid regions are more vulnerable to changes in the timing of wet and dry spells, which profoundly impacts agriculture, ecosystems, and water resource management. A shift in the timing of wet and dry spells can lead to significant challenges in food production and water resource allocation and management. Moreover, a growing body of evidence confirms that global warming can create or intensify compound climate extreme events, leading to more significant impacts on hydro-ecological systems than individual extremes alone^32^. Despite a wide range of extreme compound events, drought, coupled with heat waves, is a typical example of compounding extremes in arid and semi-arid regions^33,34^. Confirming the importance of assessing such hydro-climate extremes for decision making, precipitation and temperature trends have been studied simultaneously by recent literature^35–40^. The dry spells become more impactful during warmer periods with higher evaporation rates, intensifying the impact of drought on characteristics of hydrological stores (soil moisture, surface water, and groundwater). Temporal^41,42^, as well as spatiotemporal^43–45^ patterns studies, were specifically essential to examine the response of hydrological stores to drought conditions. Higher temperatures during wet spells lead to more rainfall than snow, while wet spells potentially cause more intense runoff during colder months. To date, very little attention has been paid to the spatial and temporal trends of temperature and precipitation associated with dry and wet spell patterns^46–48^, and no comprehensive studies have focused on the changes and comparison of the decadal trends and shifts of the mentioned indices on the entire country of Iran.

This study focused on the spatiotemporal trends of timing, duration, and occurrence of wet/dry spells and temperature in Iran. A statistical analysis approach was applied to quantify the changes in three temporal scales, including long-term (60-year), short-term (30-year), and decadal, contrasting the current literature to detect precipitation trends over time^49–52^.

## Data and methods

### Study area and data

Iran covers an area of 1.65 million square kilometers in Southwest Asia. Arid and semi-arid regions like Iran have an extremely continental climate with warm and dry summers and very cold winters, especially in the central regions. Iran is divided into 12 climate types (i: Caspian mild and wet, ii: Caspian mild, iii: Mediterranean with spring rains, iv: Mediterranean, v: cold mountains, vi: very cold mountains, vii: cold semi-desert, viii: hot semi-desert, ix: dry desert, x: hot dry desert, xi: hot coastal dry and xii: coastal dry)^53^. In this study, climate zones with similar features were divided into five categories as wet (i and ii), Mediterranean and mountain (iii, iv, v and vi), semi-desert (vii and viii), desert (ix and x), and coastal dry (xi and xii) (Fig. S1).

The daily climate data (i.e., daily precipitation, minimum and maximum temperature) from 1989 to 2018 were obtained from the Meteorological Organization of Iran to quantify the short-term to long-term changes in wet/dry spells in the selected stations^27,54,55^. We employed 30-year (1989–2018) and 60-year (1959–2018) data, respectively, for 49 and 28 synoptic stations across Iran (Table S1).

### Definition of wet/dry spells

A period of d consecutive rainy or dry days with daily rainfall amounts resulting above or below a fixed threshold is defined as a wet or dry spell. These thresholds in recent studies fall into two categories^56^, a constant value^57,58^ and variable values evaluated based on percentiles^59^ or mean daily rainfall^60^. In the present study, wet or dry spell duration is estimated based on a threshold of 0 mm/d, and therefore, a wet spell is defined as consecutive days when precipitation (AR > 0), and dry spell is defined as consecutive days with no precipitation (AR = 0).

### Definition of indices

A total of eleven indices for precipitation patterns and six for temperature were employed to study the characteristics of wet and dry spells (Table 1). The definition of indices was partially inspired by other previous studies^61,62^, but mainly based on the climate condition of the arid, semi-arid country of Iran. The trends of these indices were analyzed for three temporal periods, including decadal, 1989–2018 (30-year), and 1959–2018 (60-year), to investigate short-term to long-term spatiotemporal changes in precipitation patterns. Due to world economic growth in the 1960s, climate data during 1959–2018 and 1989–2018 were evaluated to monitor the short-term and long-run variations in the selected stations after the industrial revolution^63^. The analysis was done on two spatial scales, (i) the area of five different climates and (ii) the provincial level. The country consists of 30 provinces; based on the available data, the provincial-level analysis was done for 28 stations (Qom and Ardebil provinces were excluded due to lack of data).

**Table 1:** Dry/wet spells indices used in this study.

No	Indices	Descriptive name	Definition	Unit
1	NWS	Number of wet spells	Number of wet spell period at each year	Number
2	MWSL	Maximum wet spell length	Maximum wet spell period at each year	d
3	NDS	Number of dry spells	Number of dry spell period at each year	number
4	MDSL	Maximum dry spell length	Maximum dry spell period at each year	d
5	NDD	Number of dry days	Number of day without precipitation	d
6	NWD	Number of wet days	Number of day with precipitation	d
7	AWSL	Average wet spell length	Annual average of wet spell length	d
8	ADSL	Average dry spell length	Annual average of dry spell length	d
9	ARD	Annual rainfall depth	Cumulative rainfall depth for each year	mm
10	TMWS	Timing of maximum wet spell	Date of Maximum wet spell period	d
11	TMDS	Timing of maximum dry spell	Date of Maximum dry spell period	d
12	MWSMXT	Maximum wet spell max temp	Maximum temperature during Maximum wet spell period	°C
13	MWSMT	Maximum wet spell mean temp	Mean temperature during Maximum wet spell period	°C
14	MWSMNT	Maximum wet spell min temp	Minimum temperature during Maximum wet spell period	°C
15	MDSMXT	Maximum dry spell max temp	Maximum temperature during Maximum dry spell period	°C
16	MDSMT	Maximum dry spell mean temp	Mean temperature during Maximum dry spell period	°C
17	MDSMNT	Maximum dry spell min temp	Minimum temperature during Maximum dry spell period	°C

### Linear tendency test

The well-known non-parametric Mann–Kendall (MK)^64,65^ test was applied to compute the linear tendencies. This method of detecting monotonic trends, is distribution-free and rank-based, with the advantage of having minimal assumptions for time series. This method used a statistical parameter (Z) to represent the trend significance test. The Mann–Kendall rank trend test statistic, Z, is computed based on the following equation:

1 $$Z = \left\{ {\begin{array}{*{20}l} {\frac{S - 1}{{\sqrt {{\text{var}} (s)} }}\;\;\;\;\;\;\;S > 0} \\ {0\;\;\;\;\;\;\;\;\;\;\;\;\;\;\;\;S = 0} \\ {\frac{S + 1}{{\sqrt {{\text{var}} (s)} }}\quad S < 0} \\ \end{array} } \right.$$

where:

2 $$S = \mathop \sum \limits_{i = 1}^{N - 1} \mathop \sum \limits_{j = i + 1}^{N} {\text{sgn}} (x_{j} - x_{i} )$$

3 $${\text{sgn}} (\theta ) = {\text{sgn}} (x_{j} - x_{i} ) = \left\{ {\begin{array}{*{20}c} { - 1} \\ {\;\;0} \\ {\;\;1} \\ \end{array} } \right.\;\;\;\;\;\left. {\begin{array}{*{20}c} {\theta < 0} \\ {\theta = 0} \\ {\theta > 0} \\ \end{array} } \right\}$$

If N > 10, the variance is computed as:

4 $$Var\left( s \right) = \frac{{N\left( {N - 1} \right)\left( {2N + 5} \right) - \mathop \sum \nolimits_{i = 1}^{P} t_{i} \left( {t_{i} - 1} \right)\left( {2t_{i} + 5} \right)}}{18}$$

Else, if N ≤ 10, the variance is computed as:

5 $${\text{var}} (s) = \frac{{N\left( {N - 1} \right)\left( {2N + 5} \right)}}{18}$$

in which the x_j_ and x_i_ are the sequential data values (j > i), N is the length of the data set, P shows the number of tied groups, t is the extent of any given tie, and Σ denotes the summation over all ties. A positive value for Z shows an increasing trend in the time series, whereas a negative value shows a decreasing trend.

The Mann–Kendall test has the null hypothesis (no trend in time series). The region of rejection of the standardized test statistic, Z, depends on the specification of the significance level α.

Null hypothesis is rejected and a significant trend exists in the time series if $\left| Z \right| > Z_{1 - \alpha /2}$, in which $Z_{1 - \alpha /2}$ is obtained from the standard normal distribution table. In this study, the statistical significance of the trends was assessed at a 95% confidence level (*P*_value_ < 0.05) if |Z|≤ 1.96.

To eliminate or reduce the influence of serial correlation on the Mann–Kendall test, pre-whitening process has been suggested to be applied for time series analysis^66^.

The following procedure is used to examine the possible statistically significant trends in sample data $\left( {x_{1} ,x_{2} ,...,x_{n} } \right)$:

*Step 1-* The lag-1 serial correlation coefficient of sample data x_i_ (designated by r_1_) is computed as follows^67^:

6 $$r_{1} = \frac{{\frac{1}{N - 1}\mathop \sum \nolimits_{i = 1}^{N - 1} (x_{i} - \overline{x})\left( {x_{i + 1} - \overline{x}} \right)}}{{\frac{1}{N}\mathop \sum \nolimits_{i = 1}^{N} (x_{i} - \overline{x})^{2} }}$$

where $\overline{x }$ is the mean of sample data, and n is the sample size.

*Step 2–1-* In case of calculated $r_{1}$ being true in Eq. (7) at $\alpha = 0.05$, the Mann–Kendall test can be used.

7 $$\frac{{ - 1 - Z\left( {1 - \frac{\alpha }{2}} \right)\sqrt {N - 2} }}{N - 1} \le r_{1} \le \frac{{ - 1 + Z\left( {1 - \frac{\alpha }{2}} \right)\sqrt {N - 2} }}{N - 1}$$

*Step 2–2-* Given that the calculated $r_{1}$ isn't true in Eq. 7, data has a serial correlation which needs to be corrected by first calculating Sen’s Slope (SS) non-parametric trend estimator^68^:

8 $$\beta = Median\left( {\frac{{x_{j} - x_{l} }}{j - l}} \right)$$

where $x_{l}$ and $x_{j}$ are the data values at times j and l (j > l), respectively.

Afterwards,$y_{i}$ value is calculated by using Eqs. (9), (10) and (11):

9 $$x_{i}^{^{\prime}} = x_{i} - \left( {\beta *i} \right)$$

10 $$y_{i}^{^{\prime}} = x_{i}^{^{\prime}} - r_{1} \left( {x_{i - 1}^{^{\prime}} } \right)y_{1}^{^{\prime}} = x_{1}^{^{\prime}}$$

11 $$y_{i} = y_{i}^{^{\prime}} + \left( {\beta *i} \right)$$

Finally, the trend of $y_{i}$ is calculated by Eqs. (1)–(5).

### Change point detection

While several methods are applicable to determine change points of a time series^69–71^, the most commonly used test for change point detection is Pettitt’s test^72^. Accordingly, in this study, Pettitt’s test was applied to detect a single change-point in Iran's climate series over the past 60 years. This method tests whether a time series follows one or more distributions with the exact location parameter (no change) or whether a change point exists. In this method, a non-parametric statistic (U_t_) was defined, and the significance probability of the test was approximated for *P*_value_ < 0.05. The procedures for the non-parametric test statistics Ut is described as follows:

12 $$K_{T} = \max \left| {U_{t,T} } \right|$$

where:

13 $$U_{t,T} = \mathop \sum \limits_{i = 1}^{t - 1} \mathop \sum \limits_{j = i + 1}^{T} {\text{sgn}} (x_{i} - x_{j} )$$

If the static is significant, K_T_ is the change point of the time series. The significance probability of K_T_ for $p \le 0.05$ can be calculated as:

14 $$p \simeq 2{\text{ exp}}\left( {\frac{{ - 6 K_{T}^{2} }}{{T^{3} + T^{2} }}} \right).$$

## Results and discussion

### Overall tendencies and temporal patterns

**Figure 1:**
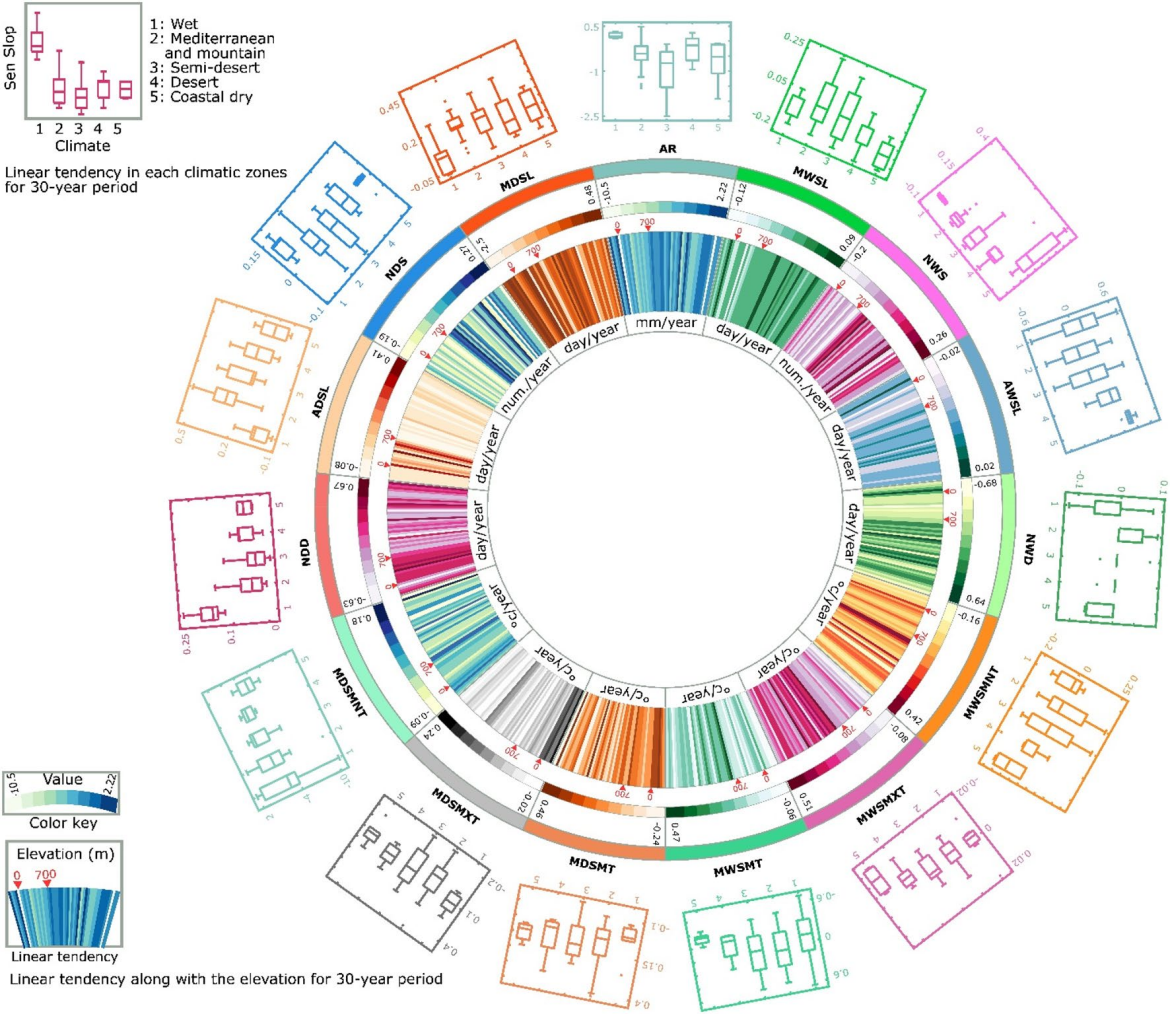
Dry/wet spells characteristics linear tendencies during decades and comparison of long-term with short-term period in Iran. *Note* The figure is produced by the authors using Circos^75^ 0.69-9 v 2019. (http://circos.ca/software/download/circos/).

During 1959–2018, the AR over Iran faced a persistent declining tendency, which intensified after 1989. The linear tendencies of AR across Iran ranged from − 4.02 to 1.61 (− 10.5 to 2.23) mm/year, with an average of − 0.5 (− 1.5) mm/year (*P*_value_ < 0.05) over the past 60 (30) years (Fig. 1). The results of the MK test indicated significant increasing trends in the AR of 61.9% of stations before the mid-1980s, and thereafter, a significant decreasing trend was seen. While 16.3% of stations had significantly decreasing trends, only 25% showed insignificant increasing trends (*P*_value_ > 0.05) during 1989–2018. According to the significant negative trends in AR, most stations have experienced an increase in drought severity, duration, and frequency over the last three decades across Iran^6,54,73,74^.

Temporal variations in precipitation features have significantly contributed to AR changes over the last 30 years, compared to the earlier study period. For instance, the NDD/NWD revealed significant decreasing/increasing trends at a rate of 0.15 days/year during the 60-year period, while developing have reversed (increasing/decreasing) and insignificant trends during the last three decades (Fig. 1).

In the past six (three) decades, the linear tendencies of NWD showed that 20.7% (41.4%) of stations had a negative trend, contrarily 65.5% (37.9%) of stations had a positive trend. It is worth mentioning that only 6.9% (41.4%) of stations indicated significant trends. For the NDD, approximately 20.7% (41.4%) of stations showed positive trends; out of these, only 10.3% (0.0%) were significant. 65.5% (37.9%) of stations indicated a decreasing trend; with 41.3% (6.8%) being significant (P_value_ < 0.05); the remaining 10.7% (17.9%) showed no trend during the 60 (30)-year period.

The analysis of wet spells indicated a dramatic decline in positive or significantly positive trends in the number of 1 to 3-day wet spells over the last 30 years compared to the last 60 years (Fig. S2). The results showed that 10.7% of stations exhibited downward trends (*P*_value_ > 0.05) in the accumulated rainfall in 2 to 5-day wet spells during 1959–2018, and more stations (25%) experienced decreasing tendencies (*P*_value_ > 0.05) over the last three decades. In the past 60 (30) years, the linear tendencies of the accumulated rainfall in 2 to 6-day wet spells ranged from − 2.86 to 0.65 (− 4.60 to 3.17) mm/year, with an overall average of -0.16 (-0.35) mm/year (Fig. S3). In contrast, the number of 1-day wet spells exhibited increasing trends and the accumulated rainfall exhibited decreasing tendencies at the rates of 0.02 (0.02) days/year and − 0.31 (− 0.15) mm/year), respectively. 67.85% (32.14%) of stations indicated positive linear tendencies in NWS in the past 60 (30) years, while the number of stations with positive trends in accumulated rainfall during 1-day wet spells doubled during 1989–2018. As Iran is characterized by infrequent, highly variable rainfall^76^, a reduction in the number of 1-day wet spells and an increase in its accumulated rainfall does not significantly influence annual rainfall throughout the country; however, it may increase the risk of flood occurrence. A similar trend has been detected in other arid and semi-arid regions of the world, where insignificant trends in AR coupled with intensified daily rainfall have increased the risk of flash floods^56,77^.

During the past 30 (60) years, 6 (3) out of 10 stations with the highest annual snowfall (Mediterranean and mountain climate zones) exhibited significant positive trends (*P*_value_ < 0.05) in MWSMXT (Table S1). The rates of increase in MWSMXT, MWSMT, and MWSMNT were 0.33 (0.08), 0.21 (0.04), and 0.06 (0.00) °C/year, respectively. This is more than four times greater (1989–2018) than the corresponding rates in 1959–2018. This might have led to a sharp decline in total snowfall due to more winter precipitation in the form of rain instead of snow, which is consistent with the observed changes in recent decades.

### Association between climate and wet/dry spells pattern

There was a slight upward trend in the overall temperature during MDSL (*P*_value_ < 0.05) and MWSL (*P*_value_ > 0.05) when we compare long-term data to that of more recent years, and of the different climates, the increase was greater in the wet climate zone. In the longer study period (1959–2018) changes for MDSMT and MWSMT were 0.06 and 0.03 °C/year, while in the shorter study period (1989–2018) these figures almost doubled (Fig. 1 and Table S1). The MDSMXT showed an increasing trend at a rate of 0.04 (0.18) °C/year (*P*_value_ < 0.05) over the past 60 (30) years in the wet climate zone and the recent linear tendencies ranged from 0.15 to 0.24 °C/year. The results showed that the MDSMT also followed upward trends at a rate of 0.06 (0.11) °C /year ranging from 0.04 to 0.10 (0.04–0.13) °C/year (*P*_value_ < 0.05) over the past 60 (30) years in this climate zone. Despite the small area (3.5%), the wet climate zone contributes considerably to the annual rainfall depth in Iran^78^. The increasing temperature was expected to increase evaporation rates, leading to higher humidity during the summer in this climate zone. Moreover, the significant increase in MDSL and its temperature, coupled with rainfall reduction, has resulted in drier and warmer climate conditions in the last 30 years compared to the past 60 years, consistent with the previous studies^79^.

### Decadal comparisons in wet and dry spells indices

To address more temporally detailed changes in wet and dry spells, the indices were examined on a decadal scale (i: 1959–1968, ii: 1969–1978, iii: 1979–1988, iv: 1989–1998, v: 1999–2008 and vi: 2009–2018). Generally, the results indicated that NWS (0.43, 0.17, 0.07, 0.55, 0.26 and − 0.39 number/year for decades i-vi, respectively), NWD (1.2, 0.28, 0.31, 1.41, 0.97 and − 0.48 days/year for decades i-vi, respectively), and MWSMNT (0.26, 0.18, 0.18, 0.47, − 0.51 and − 0.14 °C/year for decades i-vi, respectively) experienced a reversed (declining) trend in the last decade (Fig. 1). However, decreasing trends for NDD (− 1.21, − 0.28, 0.28, − 1.13, − 0.97 and 0.49 days/year for decades i-vi, respectively) and ADSL (− 0.10, − 0.16, 0.12, − 0.22, − 0.09 and 0.09 days/year for decades i-vi respectively) during the 1959–1968 and 1999–2008 periods have also reversed in the last decade. The increasing dry spells indices (except NDS) were found in the last (2009–2018) decade. Therefore, AR (1.74, 2.28, -0.01, 0.08, 1.47 and − 0.26 mm/year for decades i-vi, respectively) has increased throughout the first four decades, while the rate of change has switched to a negative value over the last decade.

Iran has encountered a decrease in AR, ranging from − 37.7 to 46.16 mm, with an overall average of − 0.26 mm in 2009–2018 (Fig. 1). This may be due to (1) sudden reductions in the NWS and NWD (− 0.65 number/year and − 1.45 days/year), (2) abrupt increases in NDD (1.46 days/year), or (3) sudden increases in ADSL (0.18 days/year) and MDSL (0.73 days/year) during this decade. The increase in MDSL (1.49 days/year), NDD (0.49 days/year) and ADSL (0.09 days/year) caused a significant (*P*_value_ < 0.05) decline in NDS (-0.33 days/year) in the last decade.

The results of MWSMNT (0.26, 0.18, 0.18, 0.47, − 0.51 and − 0.14 °C/year for decades i-vi, respectively), MWSMXT (0.34, 0.14, 0.16, 0.54, − 0.43 and 0.78 °C/year for decades i-vi, respectively), MWSMT (0.31, 0.13, 0.28, 0.41, − 0.37and 0.29 °C/year for decades i-vi, respectively), MDSMNT (0.15, 0.17, − 0.07, 0.34, − 0.10 and − 0.91 °C/year for decades i-vi, respectively), MDSMXT (0.00, 0.16, − 0.24, 0.00, 0.09 and 0.06 °C/year for decades i-vi, respectively), and MDSMT (0.07, 0.16, − 0.06, 0.16, − 0.04 and -0.31 °C/year for decades i-vi, respectively) indicated that Iran has generally experienced warmer (wet and dry) spells over the past decades (Fig. 1). The accumulated rainfall in 4 to 6-day wet spells ranged from − 38.9 to 17.24 mm/year with average of − 0.48 mm/year during the 2009–2018 period, greater decreasing rates than 1999–2008 (− 16.2 to 35.83 mm/year with an average of 0.7 mm/year) especially in stations located below sea level.

Generally, saturation vapor pressure increases more rapidly as temperature increases, leading to a higher capacity to contain water vapor than cold air. Therefore, the increase in temperature meant that dry regions (major parts of Iran) tended to have much less annual rainfall due to decreased NWD and NWS. Although AR decreased in the wet climate zones like the Caspian Sea coastline, increasing water vapor content under global warming increased extreme short-term precipitation in the last two decades, which agrees with the previous studies and recent heavy and super-heavy rainfall^80^.

It can be concluded that most detected trends in climatic patterns across Iran have started in the last two decades (1999–2008 and 2009–2018) and become more severe in the last decade (2009–2018); this can be attributed to anthropogenic climate change. Moreover, stations with lower elevations, mostly located near sea level, seemed to be more affected.

### Regional tendencies and spatial patterns

**Figure 2:**
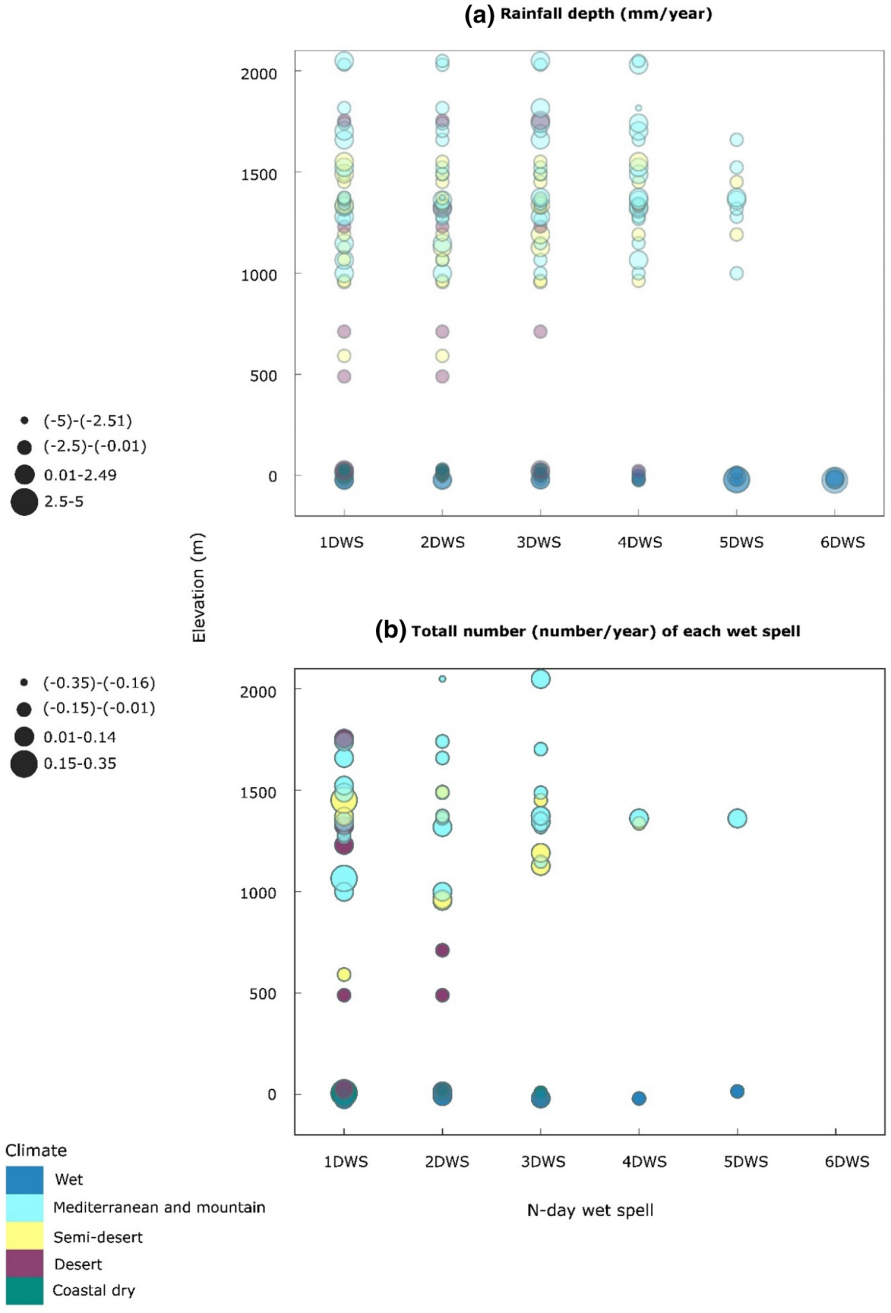
Changes of (**a**) rainfall depth (mm/year) and (**b**) total number (number/year) of each wet spell along with elevation.

To understand the dramatic decline in AR during 1989–2018, the trends in temperature and wet/dry indices have been studied more specifically with a higher number (49) of stations, nearly double the previous number (28 stations), to see whether they were connected with changes in climate or elevation. The MK test showed that the linear tendencies of AR in the past three decades ranged from − 10.50 to 2.23 mm/year, with an average of − 2.04 mm/year. The results also show that 83.67% of stations followed decreasing trends in AR, and only 16.33% of stations showed increasing trends during 1989–2018. Meanwhile, 16.33% of stations experienced significant decreasing trends (*P*_value_ < 0.05), and no stations exhibited significant increasing trends (*P*_value_ < 0.05) in AR across Iran. The results also indicated that the linear tendencies of annual rainfall accumulated in 1–6-day wet spells ranged from − 4.61 to 3.17 mm/year, with an average of − 0.28 mm/year (*P*_value_ < 0.05) (Fig. 2b). In contrast, the total number of each n-day wet spells has decreased at lower rates (− 0.33 to 0.27 number/year) over the past 30 years (Fig. 2a).

#### Impacts of latitude, longitude, and altitude on pattern changes

In the past three decades, all climate zones in Iran revealed a relatively similar (primarily downward) trend in AR. However, there was no general pattern in the AR changes in wet climate zones, where changes varied between − 10.5 and 2 mm/year. Therefore, considering how factors such as altitude and distance from water bodies is vital for interpreting the observed changes and reaching a conclusive attribution of the observed changes to either of these factors.

**Figure 3:**
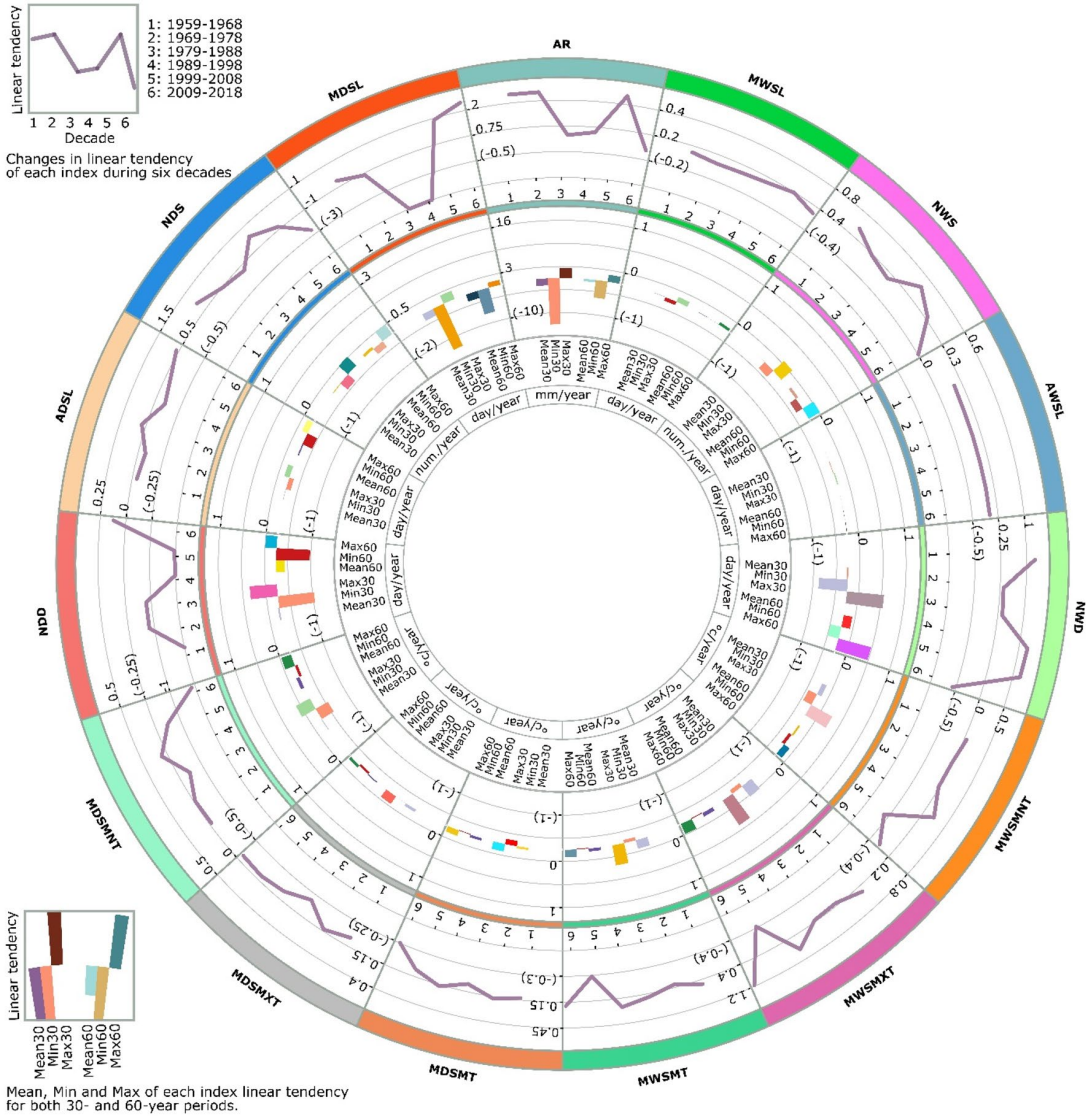
Dry/wet spells characteristics linear tendencies in various temporal scales and climate zones in Iran. *Note* The figure is produced by the authors using Circos^75^ 0.69-9 v 2019. (http://circos.ca/software/download/circos/).

Iran is surrounded by water bodies in the north, the Caspian Sea, and south, the Persian Gulf and the Oman Sea. According to the spatial pattern of AWSL (ranging between − 0.02 and 0.02 days/year) and MWSL (ranging between − 0.12 and 0.09 days/year), the maximum decreasing trend stations were observed near these water bodies (e.g., Gharakhil, Siri, Bandarelenge, Gorgan and Babolsar). However, most stations indicating significant decreasing trends in the mentioned indices, as well as AR, were near southern Iranian water bodies. Meanwhile, in terms of temperature indices, the highest decreasing trends in both MWSMXT ((− 0.08)–0.18 °C/year) and MDSMT ((− 0.06)–0.25 °C /year) occurred in northern regions (Fig. 3).

The results revealed that increasing MWSMT and MWSMXT appeared to have intensified with increasing distance from water bodies. MWSMT and MWSMXT have risen dramatically in the Mediterranean and mountain (0.19 and 0.29 °C/year), semi-desert (0.15 and 0.22 °C/year) and desert (0.12 and 0.18 °C/year) climate zones. Following the highest trend rates, the MWSMT and MWSMXT in the Mediterranean and mountain zone (e.g., Mashhad, Bojnourd and Tabriz stations) experienced increasing rates, three times greater than stations adjacent to water bodies (e.g., Rasht, Ramsar, Kish and Bandareabbas). According to the considerable amount of snowfall in Mediterranean and mountain zones, this warming in wet spells might have been the main reason for the change in winter precipitation from snow to rainfall, showing agreement with similar studies^81^.

Although the stations near the southern and northern water bodies showed the same trends in the analyzed indices, they are located in two different climate zones^53^. The most significant decrease of AR trend, at an average rate of − 3.11 (− 5.14 to − 0.96) mm /year, was found in stations located in southern Iran with a coastal hot desert climate. Increasing NDD and dry spell length coincides with reductions in the AWSL, and NWD can increase the risk of heavy rainfall in the southern Iranian coastal area. In contrast, almost all the stations in northern Iran (wet climate) were characterized by a slight decreasing (increasing) trend of AR in 71.4% (28.6%) of stations, and most of the stations in these regions exhibited an insignificant trend with an average rate of − 2.42 ((− 10.50) to 2.23) mm/year over recent decades.

Among all coastal stations, the most significant increase in MDSMXT (0.17 °C /year) was calculated at an average rate of 0.17 (0.02–0.07) °C /year for stations near the Caspian Sea coastline in the last 30 years. While coastal dry zones (Southern water bodies) experienced linear tendencies ranging from 0.02 to 0.07 °C/year (Fig. 3). Higher MDSMXT has intensified the evaporation rates from water bodies, leading to more precipitation, likely caused by climate change. This may be the main reason for relatively milder linear tendencies of AR reduction in northern coastal areas (-2.42 mm/year) with wetter climates compared to stations located near southern water bodies (-3.11 mm/year) with a coastal dry climate.

Generally, the lower elevations showed greater changes in accumulated rainfall in 2 to 6-day wet spells over recent decades. According to Fig. 2b, the accumulated rainfall in 4-day wet spells showed a decreasing trend of − 1.5 (− 1.99 to − 0.09) mm/year at elevations below sea level, while this rate was about − 0.24 (− 3.49 to 0.63) mm/year at higher elevations. Meanwhile, low altitude stations (below mean sea level) had insignificant increasing trends, with an average rate of 0.7 (− 3.11 to 2.7) mm/year in the 5-day accumulated rainfall, while this trend was reversed by (− 0.11) mm/year ranging from − 1.74 to 0.62 mm/year for stations above the sea level. Despite having no changes in the total number of 6-day wet spells, stations located below sea level exhibited an upward trend (*P*_value_ > 0.05) in their rainfall accumulated, with an average of about 1.15 mm/year; This matches the observed rainfall pattern and recent floods in these areas^82^.

The ADSL and the NDD had significant positive correlations with altitude at stations located at less than 700 m (*P*_value_ < 0.05, N = 17), while a negative correlation with altitude was detected at stations higher than 700 amsl. Generally, the NWD and MWSL increased, but MDSL has decreased more intensely with elevations across Iran.

The overall results indicated that AR was falling across Iran, but the AR has decreased more intensely with decreasing altitude. Three possible reasons exist for this (Fig. 3) increasing tendencies of temperature within MDSL (0.08 °C /year)/MWSL (0.08 °C /year); (2) decreasing trends in wet spell indices (i.e., AWSL (− 0.01 days/year), NWD (− 0.2 days/year)); and (3) increasing tendencies in dry indices (i.e., ADSL (0.09 days/year), NDD (0.21 days/year)) with decreasing elevations. Several studies have documented the relationships between temperature and topography (elevation) and their effects on rainfall over the last 30 years in Iran^7,9,30,46^. This is consistent with the observational results of the present study. However, Ahmadi et al. (2018) have suggested a reverse relationship between elevation and rainfall across Iran^83^.

MDSL and MWSL have shifted towards later timings from 1989 to 2018, but the linear tendency for TMWSL (0.53 day/year) was four times higher than TMDSL (0.14 day/year). The results indicated that TMWSL started at a later time in all climate zones. The maximum and minimum shifts in TMWSL were calculated for coastal dry (around 0.08 day/year) and desert stations (0.9 day/year), respectively. In the latter climate, the TMDSL occurred about 0.25 day/year sooner in 1989–2018. The experienced changes in TMDSL and TMWSL could significantly affect climatic patterns and socioeconomic activities, especially in agricultural sector (Fig. S5).

#### Provincial analysis

**Figure 4:**
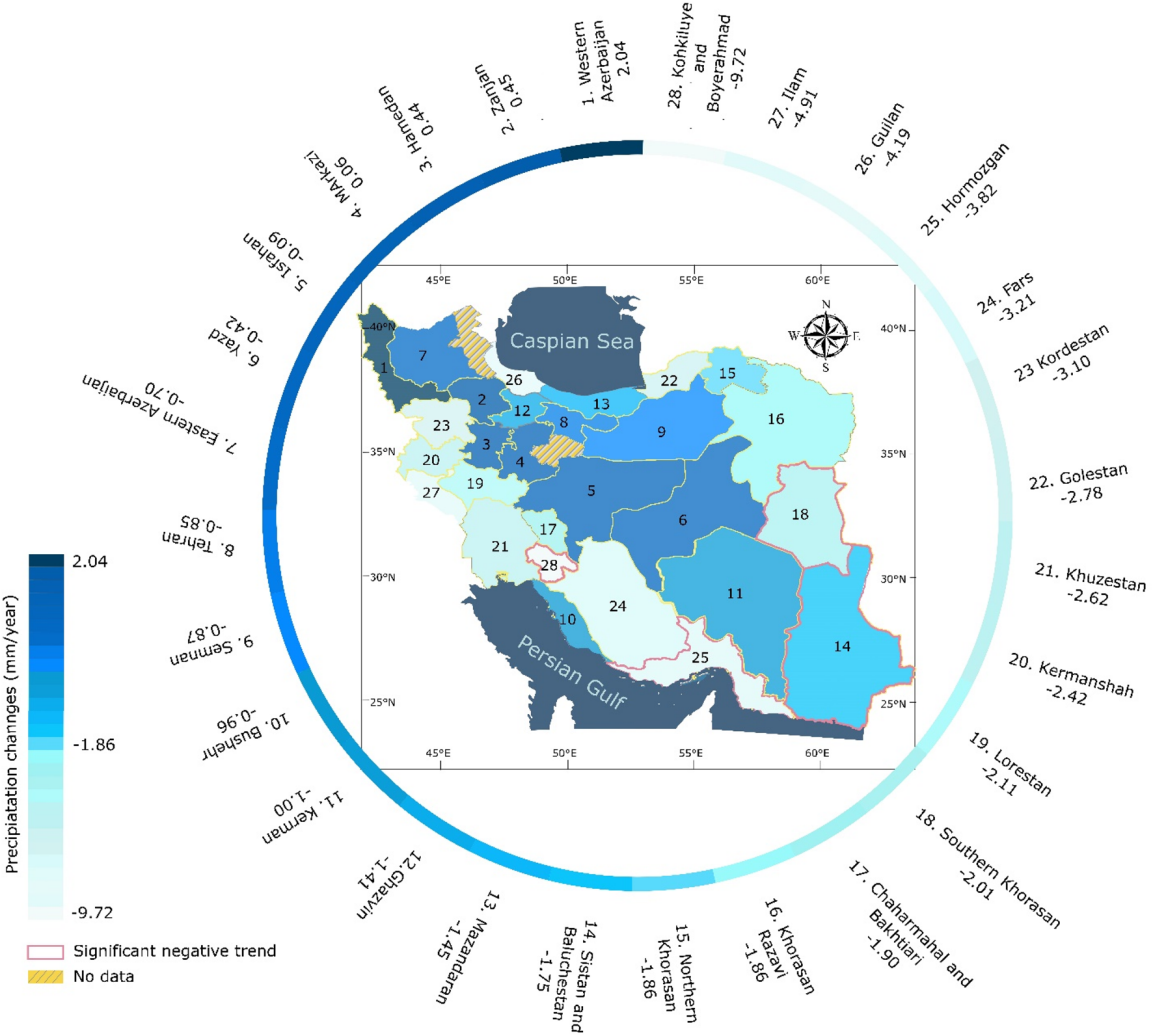
Spatial pattern of precipitation changes (mm/year) in Iran (The boarder of provinces with significant negative trend is colored red) (No province had a significant positive trend) (Qom and Ardabil provinces were excluded due to a lack of data). *Note* The figure is produced by the authors using QGIS 3.01 v 2018. (https://www.qgis.org/en/site/forusers/download.html).

At the provincial level, 24 (out of 28) provinces showed significant negative trends (*P*_value_ < 0.05) in AR (ranging from − 0.09 to − 9.71 mm/year); in contrast, for four (one) provinces, there was a positive (significant) trend ranging from 0.06 to 2.03 mm/year (Fig. 4). The Kohkiluyeh and Boyerahmad, Ilam, and Guilan Provinces had the most prominent AR decline, ranging from − 9.71 to − 4.19 mm/year. In contrast, Western Azerbaijan was characterized by an increasing tendency (*P*_value_ < 0.05) at a rate of 2.03 mm/year, while Zanjan, Hamedan, and Markazi Provinces exhibited insignificant (*P*_value_ > 0.05) upward trends, ranging from 0.06 to 0.45.

The provinces located in western and southwestern Iran experienced the highest declines in AR, ranging from − 9.72 to − 1.90 mm/year over the last three decades. Likewise, Golestan and Guilan Provinces located in Northern Iran with wet climates were characterized by significant reductions in AR at the rates of − 2.78 and − 4.19 mm/year, respectively.

## Conclusion

The characteristics of wet/dry spells were examined using extensive datasets from 49 stations across Iran. The results revealed an ongoing downward trend in AR over the past six decades and a more intense decline over the past 30 years, with no station exhibiting significant increasing trends across Iran. These major ongoing tendencies in climatic patterns in Iran started in the last two decades and have reached their highest levels of severity from 2009 to 2018.

Increasing saturation vapor pressure under warming dry/wet spells was the main reason for lower annual rainfall, due to decreased NWD and NWS in recent decades. Such hydro-climate anomalies were likely caused by anthropogenic warming and associated changes in the hydrologic cycle. Although AR decreased in wet climates at the rate of (− 2.42 mm/year), like the Caspian Sea coastline, increasing water vapor content under global warming increased extreme short-term precipitation in the last two decades.

Higher evaporation rates due to the recent increase in MDSMXT has resulted in relatively milder AR reduction in northern coastal areas, with a wetter climate compared to southern coastal areas with a coastal hot desert climate. With increasing distance to water bodies, the MWSMT and MWSMXT have increased to more than three times those of areas adjacent to water bodies, altering winter precipitation patterns from snow to rainfall in Mediterranean and mountain climate zones. The results also showed that AR had decreased more intensely with decreasing elevations due to warming dry/wet spells, decreasing AWSL/NWD, and increasing tendencies in ADSL/NDD.

At a regional scale, AR in Iran generally declines from north to south and west to east, except for four provinces out of 28. Nevertheless, southern, southwestern, and some northern (Golestan and Guilan) stations are on the frontline of annual rainfall reduction, with a remarkable 9.42 mm/day decrease in one province. Shifts in the timing of MWSL as a socially relevant climate characteristic also showed not very encouraging results. TMWSL, with its profound role in socio-economic activities, has started on average 0.53 day/year later in all regions across Iran from 1989. However, the highest shift of 0.9 day/year in desert stations could leave them in a precarious situation regarding agricultural activities. The study has also shown that the trends' significance and magnitude have varied greatly in the studied periods, suggesting high‐frequency variability during 1999–2018 as the most pronounced period. Due to climatic changes, the associated changes confirm the alteration of dry/wet spell features in Iran. The expected increase in air temperature under climate change would result in further dry and warm conditions in Iran in the coming decades. Thus, defining adaptation policies is vital for coping with the negative impacts of climate change on hydro-ecological systems. Our analyses emphasize the robustness of the employed hypothesis testing method that corrects the serial dependence in precipitation and temperature time series and uses less-restrictive, non-parametric statistical techniques to evaluate the significance of trends and abrupt changes. At the same time, the existence of some limiting factors may have undermined the given results. First, relatively small number of gauge datasets (49 stations for the whole study area) is associated with some uncertainties in providing a comprehensive large-scale pattern for the occurrence of wet and dry spells. Second, fairly short record length (30 years) in some selected stations may have constrained more accurate statistics, featuring the necessity of longer precipitation records in more stations. Still, expanding knowledge about the main drivers of the studied trends is vital for formulating effective strategies for adapting or mitigating the negative effects of extreme climate events. Future studies can build on this research by exploring the role of temperature in exacerbating dry and wet spell intensities and the associated impacts on socio-economic activities. It is of note that to test the generality of the findings of this study different dry/wet spells thresholds needs to be defined in other geographical regions over a larger hydroclimatic area. Further research should be undertaken to define dry/wet spells threshold according to climate types of the study area (i.e., specific percentile).

## Supplementary Information

Supplementary Information.

## Data Availability

The input data used for the analysis during the current study is authorized by Iran Meteorological Organization. It is available from Iran Meteorological Organization as well as the corresponding author on reasonable request.

## Code availability

The code used for the analysis during the current study are available from the corresponding author on reasonable request.
