# Supplementary material for: Three-decade assessment of dry and wet spells change across Iran, a fingerprint of climate change
Source: Sci Rep. 2023 Feb 18;13:2888. doi: 10.1038/s41598-023-30040-0 (PMC9938875; doi:10.1038/s41598-023-30040-0)
Supplement: Supplementary file 1 — Supplementary Information. [file 41598_2023_30040_MOESM1_ESM.docx]

Supporting Material for “Three-decade assessment of dry and wet spells change across Iran, a fingerprint of climate change”

**Table of Contents**

S1 Overall tendencies and temporal patterns

S2 Association between climate and wet and dry spell patterns

S3 Change points in dry/wet spell time series

**Tables:**

Table S1: The characteristics of selected rain gauges across Iran

Table S2: The median of TMDS and TMWS linear tendencies over the past 30 and 60 years in selected rain gauges across Iran

Table S3: The median of TMDS and TMWS linear tendencies over the past 30 years (1989-2018) across Iran

**Figures:**

Figure S1: Climate Zones and location of stations (Stations with red circle are those with 60 years of data)

Figure S2: Changes in the number of each wet spell along with the elevation

Figure S3: Changes of accumulated rainfall for each wet spell along with the elevation

Figure S4: Temporal changes of (A) MDSL timing and (B) MWSL timing in Iran for both 30- and 60-year time periods

Figure S5: Temporal changes of MDS and MWS timing over the last three decades in Iran

Figure S6: The detected change points in 1 to 6-day wet spell time series

Figure S7: The detected change points in the accumulated rainfall during 1 to 6-day wet spell time series

**S1 Overall tendencies and temporal patterns**

Over the past 60 years, the TMDSL/TMWSL analysis indicated positive tendencies with higher rates in the recent 30 years (Fig. 1). Therefore, MDSL and MWSL have shifted to later starting dates (i.e., towards later in summer/winter). TMDSL shifted from -0.8 to 0.43 (-1.81 to 1.67) day/year, with an average of 0.07 (0.12) day/year in the past 60 (30) years (Fig. 2A). Generally, MDSL started between mid-spring and mid-summer in Iran and has shifted towards a later timing. TMWSL also increased at a rate of 0.23 (0.44) days/year, ranging from -0.49 to 1.36 (-1.83 to 2.23) day/year in the last 60 (30) years (Fig. 2B). Generally, MWSL started between late fall and mid-winter, and there was a trend towards later starting dates, thus potentially shifting more towards the reproductive phase of winter crops. Over the past 60 years, 40% of stations indicated positive tendencies in TMDSL/TMWSL, which have been reversed to downward trends, leading to earlier occurrences of MDSL/MWSL in the last three decades (Table S2).

**S2 Association between climate and wet and dry spell pattern**

The MDSMNT exhibited a significant upward trend of 0.09 (0.10) °c/year (P_value_ < .05), and the linear tendencies ranged from 0.03-0.15 (0.0-0.18) °c/year over the last 60 (30) years in wet climates. This means that the night temperature has slightly increased during the summer season, which agrees with the similar studies carried out in different parts of Iran^57, 58, 59^.

The results also revealed that the stations located in Low (<30 masl) and high (>1000 masl) altitudes showed relatively similar tendencies (0.09/0.21 day/year and 0.06/0.24 day/year) in TMDSL/TMWSL in the past six decades (Fig. S4). In the last 30 years, the high elevations experienced linear tendencies at rates of 0.16/0.67 day/year in TMDSL/TMWSL, while low elevations experienced shifts towards later (0.03 day/year) MDSL and earlier (-0.14 day/year) MWSL timings (Table S3).

**S3 Change points in dry/wet spell time series**

Fig. S6 indicated that 17.8%, 10.7% and 14.3% of all stations indicated Change Points (CPs) in the number of 1-day, 2-day, and 3-day wet spell time series, mostly belonging to desert and coastal dry climates. However, CPs in the number of 4-day wet spells, shifted from dry to wet and Mediterranean and mountain climates. There was no CP in the number of 5 to 6-day wet spells except 6-day wet spell in Rasht station. Generally, CPs happened earlier in drier climates (especially desert and coastal dry climates) than wet climates. According to Fig. S6, CPs in 1 to 2-day wet spells accounted for 25% of stations with elevations less than 100 m. However, only 15% and 5% of stations with elevation more than 1000 m, experienced CPs in 1-day and 2-day wet spells, respectively.

Regarding the accumulated rainfall in each n-day wet spell, CP in 1-day wet spells was frequent in all climates (Fig. S7). Meanwhile, CP of the accumulated rainfall in 2-day wet spells was only detected in two stations (Rasht and Tabriz) in wet and Mediterranean and mountain climates. Accumulated rainfall in 2 to 5-day wet spells experienced no CP, while Urmia, with a Mediterranean and mountain climate, was the only station with CP in the accumulated rainfall during a 6-day wet spell.

About 40% of all stations (mostly in elevations higher than 1000 m) showed CPs in accumulated rainfall in 1-day wet spells, which means abrupt changes occurred in the pattern of this index across Iran (Fig. S7). Around 40% to 60% of stations located in elevations under 100 m experienced sharp changes in NDS, ADSL, NDD, NWS, AWSL, and NWD indices.

CPs in the precipitation indices time series (i.e., the number and rainfall depth in n-day wet spells) were detected between early 1970s and late 1990s. However, these abrupt changes in temperature indices started earlier and continued until the mid-2000s. It is worth mentioning that most of the significant changes in patterns of all the mentioned indices occurred in the 1980s. Almost all stations in wet climates (stations below the sea level) experienced sharp changes in all Max dry temperature indices, while no CP was observed in this climate zone for Max wet temperature indices.

Table S1: The characteristics of selected rain gauges across Iran (Stations with 30 and 60 years of available data are shown in black and red, respectively)

| **Station** | **Elevation** | **Climate** | **Station** | **Elevation** | **Climate** |
| --- | --- | --- | --- | --- | --- |
| Abadan | 6.6 |  | Mashhad | 999.2 |  |
| Abadeh | 2030 |  | Nowshahr | -20.9 |  |
| Ahvaz | 22.5 |  | Omidiyeh | 27 |  |
| Arak | 1702.8 |  | Qazvin | 1279.1 |  |
| Babolsar | -21 |  | Ramsar | -20 |  |
| Bam | 1066.9 |  | Rasht | -8.6 |  |
| Bandareabbas | 9.4 |  | Sabzevar | 962 |  |
| Bandareanzali | -23.6 |  | Sanandaj | 1373.4 |  |
| Bandarelenge | 22.7 |  | Saqez | 1522.8 |  |
| Birjand | 1491 |  | Semnan | 1127 |  |
| Bojnourd | 1065 |  | Shahrekord | 2048.9 |  |
| Bushehr | 8.4 |  | Shahrud | 1325.2 |  |
| Esfahan | 1550.4 |  | Shiraz | 1488 |  |
| Fasa | 1268 |  | Siri | 4.4 |  |
| Gharakhil | 14.7 |  | Tabas | 711 |  |
| Gorgan | 0 |  | Tabriz | 1361 |  |
| Hamedan | 1740.8 |  | Tehran | 1191 |  |
| Ilam | 1337 |  | Torbateheydarieh | 1451 |  |
| Iranshahr | 591.1 |  | Urmia | 1328 |  |
| Kashan | 955 |  | Yasuj | 1816.3 |  |
| Kerman | 1754 |  | Yazd | 1230.2 |  |
| Kermanshah | 1318.5 |  | Zabol | 489.2 |  |
| Khorramabad | 1147.8 |  | Zahedan | 1370 |  |
| Kish | 30 |  | Zanjan | 1659.4 |  |
| Maragheh | 1344 |  |  |  |  |

| **Climate Color Legend** | | | | | | |
| --- | --- | --- | --- | --- | --- | --- |
| Wet |  | Mediterranean and mountain | | | |  |
| Semi-desert |  | | Desert |  | Coastal dry |  |

Table S2: The median of TMDS and TMWS linear tendencies over the past 30 and 60 years in selected rain gauges across Iran

| **Station** | **1959-2018** | | **1989-2018** | |
| --- | --- | --- | --- | --- |
|  | **TMDS** | **TMWS** | **TMDS** | **TMWS** |
| Abadan | 130 | 101 | 130 | 69 |
| Ahvaz | 133 | 77.5 | 131 | 74 |
| Arak | 180.5 | 92 | 177 | 83 |
| Babolsar^*†^ | 168.5 | 218.5 | 171.5 | 241 |
| Bam^*^ | 138 | 71.5 | 139.5 | 66 |
| Bandareabbas | 118.5 | 56.5 | 114.5 | 41 |
| Bandareanzali | 193 | 269.5 | 184 | 118.5 |
| Birjand^†^ | 158.5 | 58 | 157 | 61.5 |
| Esfahan | 158 | 103 | 157.5 | 101.5 |
| Gorgan^*^ | 197 | 104.5 | 198 | 99 |
| Kerman^*†^ | 147 | 41.5 | 155 | 45 |
| Kermanshah^†^ | 155 | 57.5 | 154 | 74.5 |
| Khorramabad | 165 | 116 | 155.5 | 90.5 |
| Mashhad^*†^ | 187 | 63 | 191 | 78 |
| Urmia* | 189 | 122 | 190.5 | 117 |
| Qazvin^*^ | 203.5 | 209 | 204 | 112.5 |
| Ramsar | 194 | 111.5 | 192 | 105 |
| Rasht^†^ | 195.5 | 106 | 188 | 108 |
| Sabzevar^†^ | 175 | 46 | 171 | 64 |
| Shahrekord | 171.5 | 89.5 | 155.5 | 81.5 |
| Shahrud^*^ | 179 | 80 | 190 | 87 |
| Shiraz | 198.5 | 65.5 | 193.5 | 53.5 |
| Tabriz^†^ | 203.5 | 82.5 | 207.5 | 100 |
| Tehran^*^ | 187.5 | 115.5 | 191 | 100 |
| Torbateheydarieh^*^ | 171.5 | 56 | 172 | 56 |
| Zahedan^†^ | 162.5 | 75.5 | 143.5 | 78.5 |
| Zanjan | 204 | 100 | 154.5 | 68 |

^*^ Positive trends in TMDS have reversed to negative during the past 30 years

^†^ Positive trends in TMWS have reversed to negative during the past 30 years

Table S3: The median of TMDS and TMWS linear tendencies over the past 30 years (1989-2018) across Iran

| **Station** | **TMDS** | **TMWS** | **Station** | **TMDS** | **TMWS** |
| --- | --- | --- | --- | --- | --- |
| Abadan | 130 | 101 | Mashhad | 187 | 63 |
| Abadeh | 207.5 | 91 | Nowshahr | 184.5 | 100 |
| Ahvaz | 133 | 77.5 | Omidiyeh | 129.5 | 69 |
| Arak | 180.5 | 92 | Urmia | 189 | 122 |
| Babolsar | 168.5 | 218.5 | Qazvin | 203.5 | 209 |
| Bam | 138 | 71.5 | Ramsar | 194 | 111.5 |
| Bandareabbas | 118.5 | 56.5 | Rasht | 195.5 | 106 |
| Bandareanzali | 193 | 269.5 | Sabzevar | 175 | 46 |
| Bandarelenge | 106.5 | 47 | Sanandaj | 175 | 54 |
| Birjand | 158.5 | 58 | Saqez | 169.5 | 77.5 |
| Bojnourd | 203 | 92 | Semnan | 185 | 103.5 |
| Bushehr | 126.5 | 32 | Shahrekord | 171.5 | 89.5 |
| Esfahan | 158 | 103 | Shahrud | 179 | 80 |
| Fasa | 161.5 | 68.5 | Shiraz | 198.5 | 65.5 |
| Gharakhil | 196 | 182.5 | Siri | 107 | 42.5 |
| Gorgan | 197 | 104.5 | Tabas | 142 | 45 |
| Hamedan | 173.5 | 74 | Tabriz | 203.5 | 82.5 |
| Ilam | 148.5 | 71.5 | Tehran | 187.5 | 115.5 |
| Iranshahr | 218 | 58.5 | Torbateheydarieh | 171.5 | 56 |
| Kashan | 159.5 | 80 | Yasuj | 162.5 | 48 |
| Kerman | 147 | 41.5 | Yazd | 152.5 | 73 |
| Kermanshah | 155 | 57.5 | Zabol | 127 | 39 |
| Khorramabad | 165 | 116 | Zahedan | 162.5 | 75.5 |
| Kish | 112.5 | 61 | Zanjan | 204 | 100 |
| Maragheh | 197.5 | 66 |  |  |  |


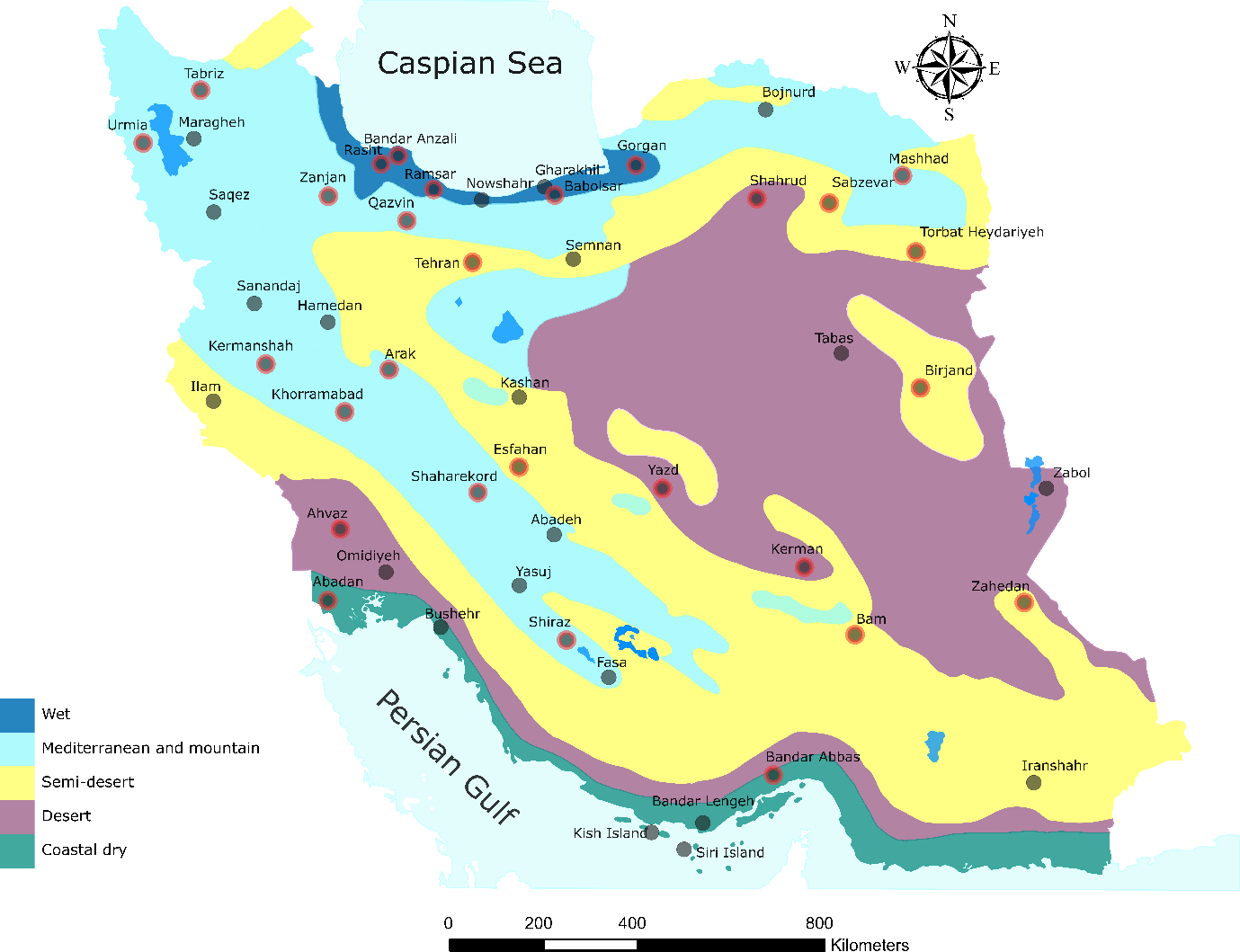


Fig. S1: Climate Zones and location of stations (Stations with red circle are those with 60 years of data) Note: The figure is produced by the authors using QGIS 3.01 v 2018. (https://www.qgis.org/en/site/forusers/download.html)


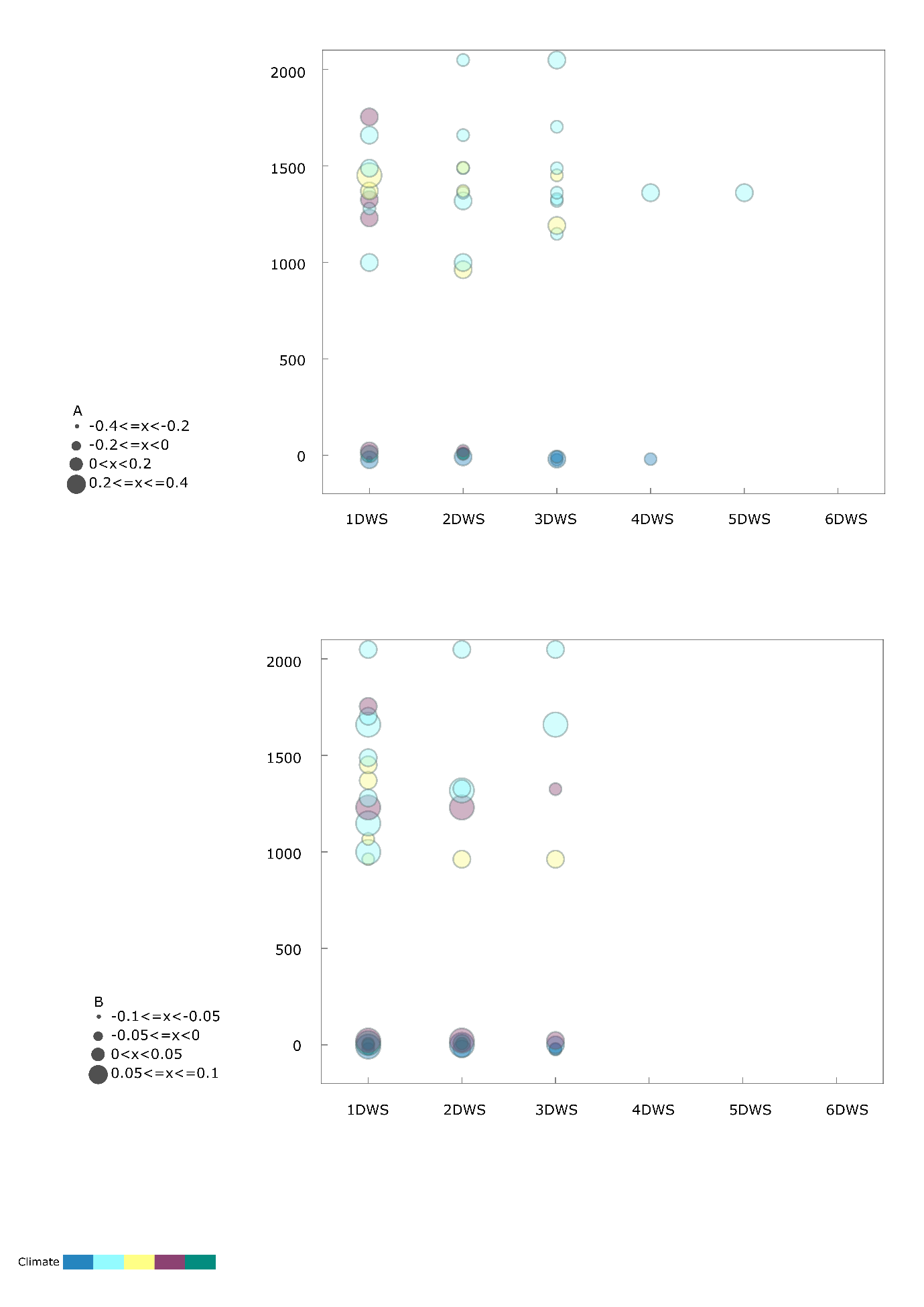


Fig. S2: Changes in the number of each wet spell along with the elevation in both 30 (A) and 60 (B) year time periods


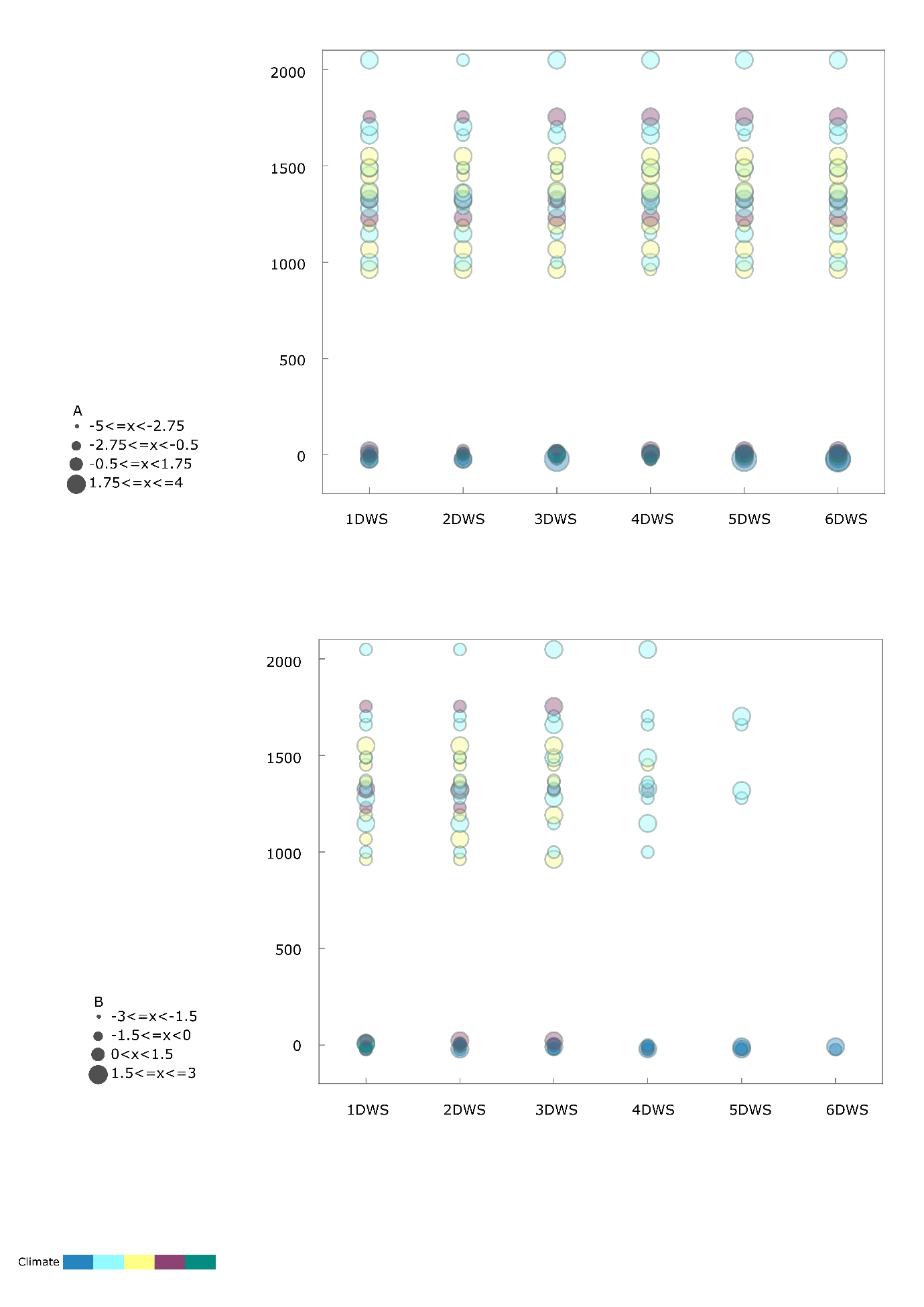


Fig. S3: Changes in the accumulated rainfall of each wet spell along with the elevation in both 30 (A) and 60 (B) year time periods

| **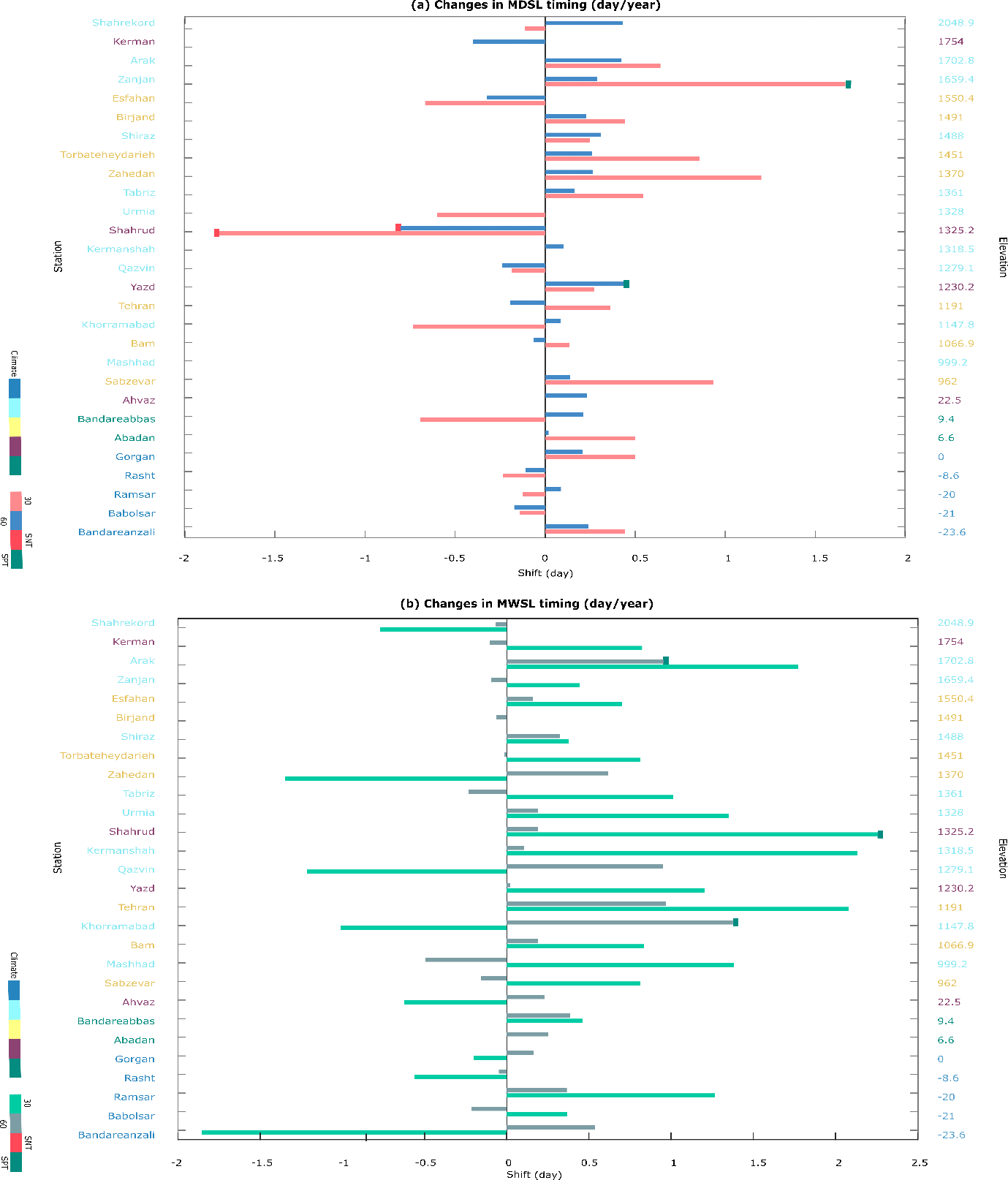** |
| --- |

Fig. S4: Temporal changes of (A) MDSL timing and (B) MWSL timing in Iran for both 30- and 60-year time periods (SN/PT=Significant Negative/Positive Trend)

**
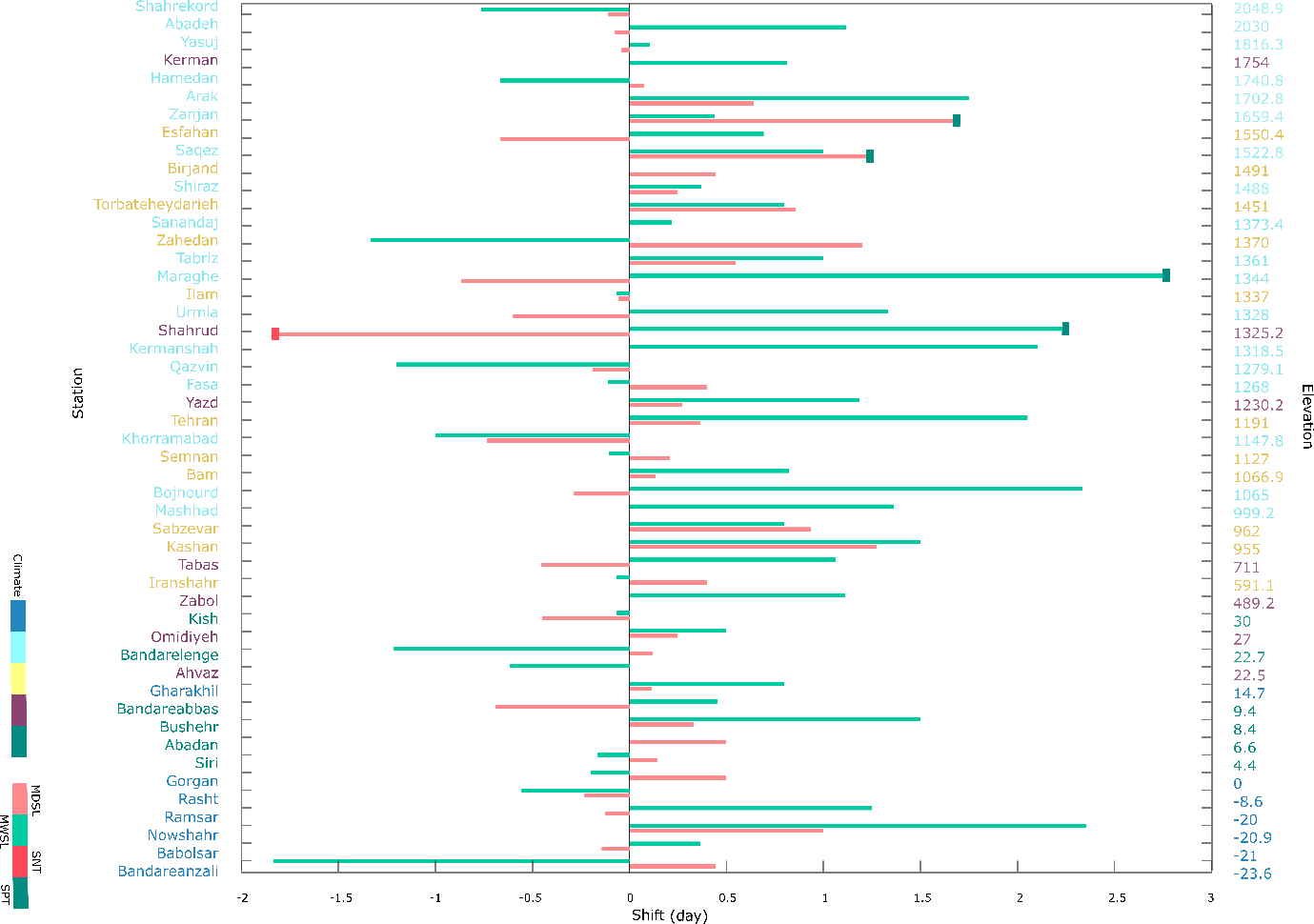
**

Fig. S5: Temporal changes of MDS and MWS timing over the last three decades over Iran (SN/PT= Significant Negative/Positive Trend)

**
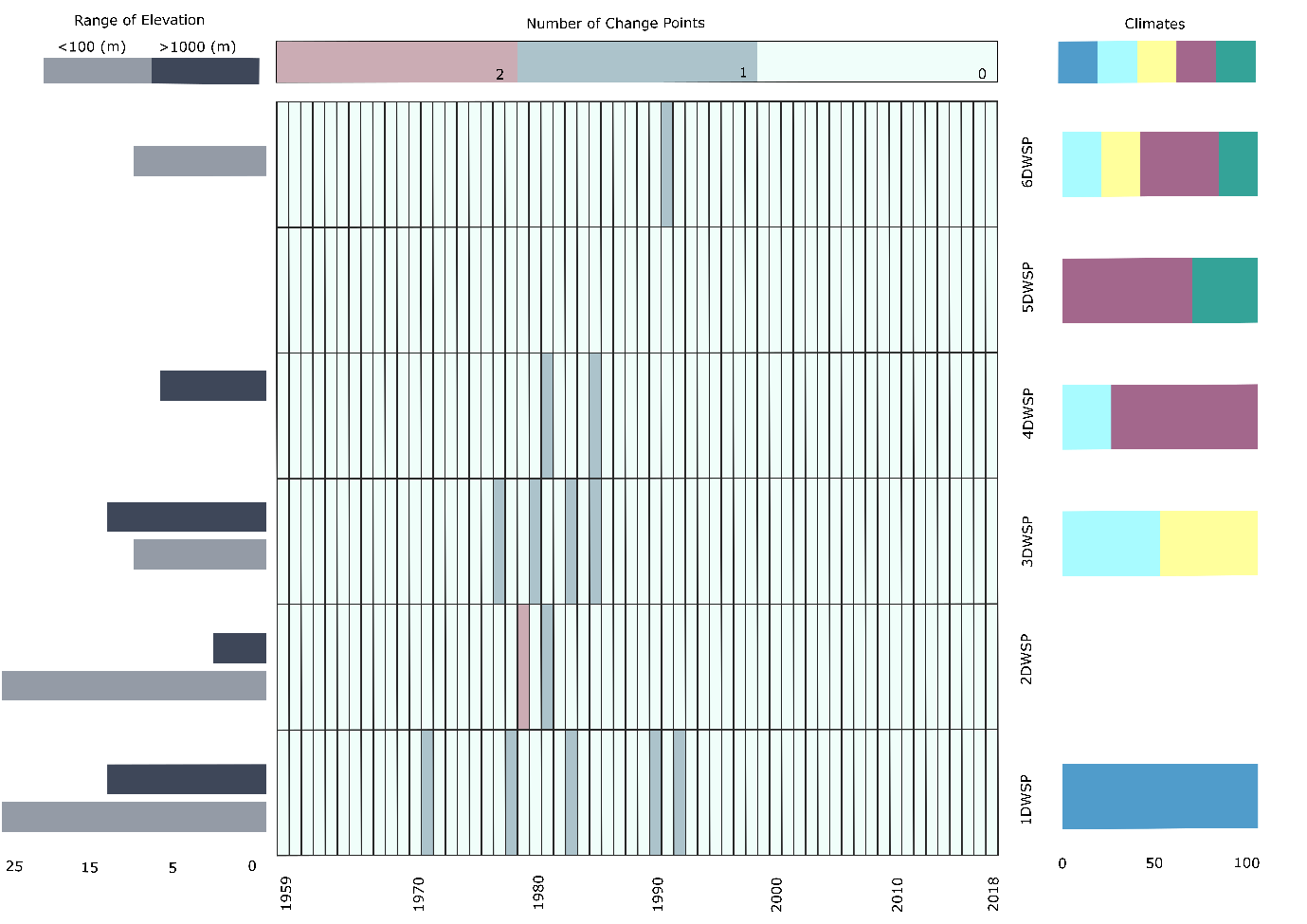
**

Fig. S6: The detected change points in 1 to 6-day wet spell time series

**
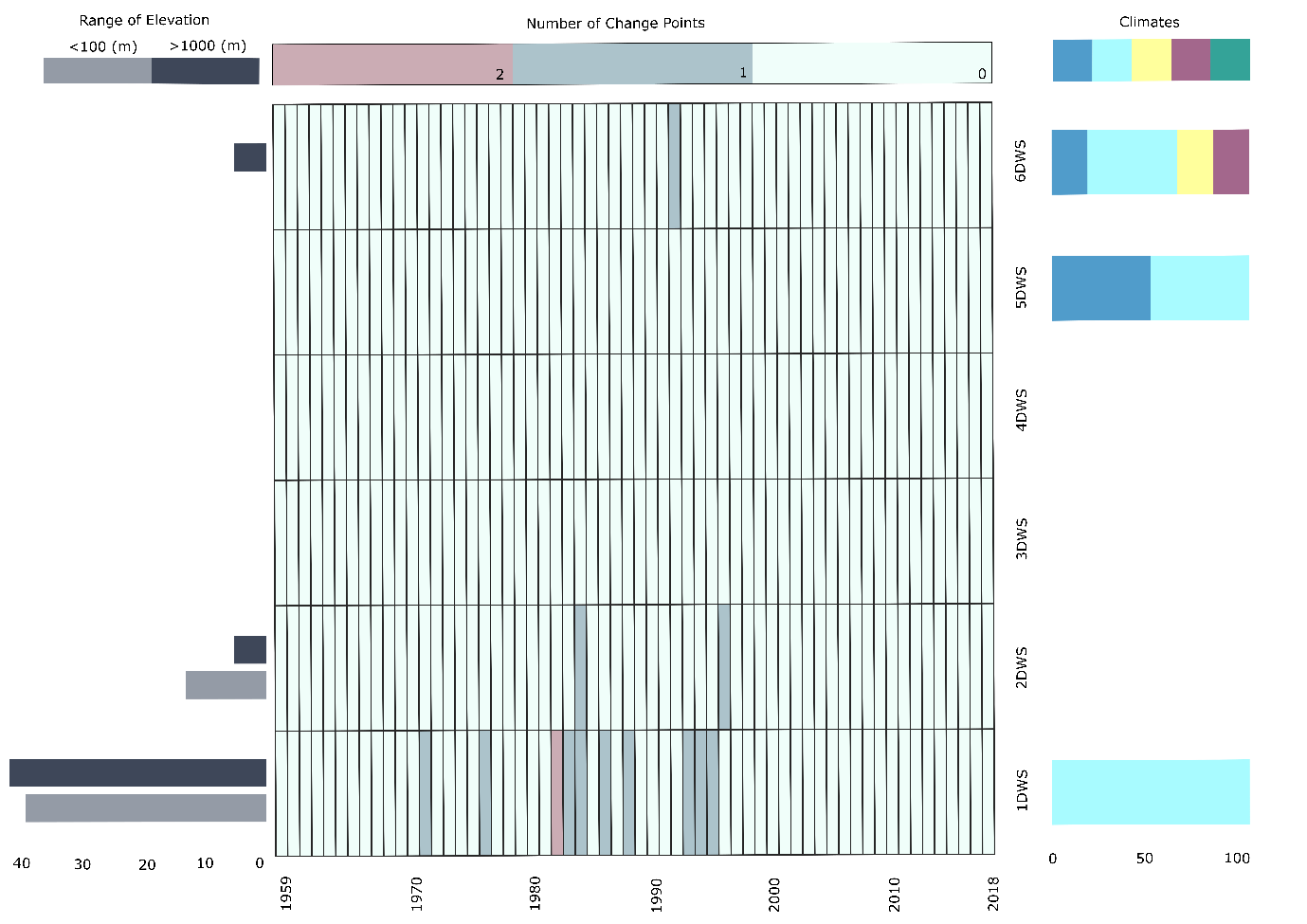
**

Fig. S7: The detected change points in the accumulated rainfall during 1 to 6-day wet spell time series
